# Tyrosol-Enriched Tomatoes by Diffusion across the Fruit Peel from a Chitosan Coating: A Proposal of Functional Food

**DOI:** 10.3390/foods10020335

**Published:** 2021-02-04

**Authors:** Silvia Tampucci, Antonella Castagna, Daniela Monti, Clementina Manera, Giuseppe Saccomanni, Patrizia Chetoni, Erica Zucchetti, Mariacristina Barbagallo, Laura Fazio, Marco Santin, Annamaria Ranieri

**Affiliations:** 1 Department of Pharmacy, University of Pisa, Via Bonanno 6, 56126 Pisa, Italy; silvia.tampucci@unipi.it (S.T.); daniela.monti@unipi.it (D.M.); clementina.manera@unipi.it (C.M.); giuseppe.saccomanni@unipi.it (G.S.); patrizia.chetoni@unipi.it (P.C.); erica.zucchetti@phd.unipi.it (E.Z.); barbagallo.mariacristina@gmail.com (M.B.); 2 Interdepartmental Research Center Nutrafood “Nutraceuticals and Food for Health”, University of Pisa, Via del Borghetto 80, 56126 Pisa, Italy; anna.maria.ranieri@unipi.it; 3 Department of Agriculture, Food and Environment, University of Pisa, Via del Borghetto 80, 56126 Pisa, Italy; l.fazio3@studenti.unipi.it (L.F.); marco.santin@agr.unipi.it (M.S.)

**Keywords:** tyrosol, chitosan, edible coatings, tomato fruit, antioxidants, carotenoids, phenolic compounds

## Abstract

Chitosan is receiving increasing attention from the food industry for being a biodegradable, non-toxic, antimicrobial biopolymer able to extend the shelf life of, and preserve the quality of, fresh food. However, few studies have investigated the ability of chitosan-based coatings to allow the diffusion of bioactive compounds into the food matrix to improve its nutraceutical quality. This research is aimed at testing whether a hydrophilic molecule (tyrosol) could diffuse from the chitosan-tyrosol coating and cross the tomato peel. To this end, in vitro permeation tests using excised tomato peel and an in vivo application of chitosan-tyrosol coating on tomato fruit, followed by tyrosol quantification in intact fruit, peel and flesh during a seven-day storage at room temperature, were performed. Both approaches demonstrated the ability of tyrosol to permeate across the fruit peel. Along with a decreased tyrosol content in the peel, its concentration within the flesh was increased, indicating an active transfer of tyrosol into this tissue. This finding, together with the maintenance of constant tyrosol levels during the seven-day storage period, is very promising for the use of chitosan formulations to produce functional tomato fruit.

## 1. Introduction

The tomato is one of the main components of the Mediterranean diet and is consumed worldwide. Its health-promoting properties are mainly due to the presence of lycopene, a carotenoid rarely found in other fruit and vegetables [1]. The tomato also contains phenolic compounds, such as hydroxycinnamic acids and flavonoids, which contribute to the nutraceutical value of this fruit. However, phenolic compounds are mainly concentrated in the peel, which represents approximately 5% of the whole fruit [2,3]. Accordingly, the interest in increasing the concentration of phenolic compounds in tomato flesh is growing. The concern of most consumers about transgenic organisms, as well as the national regulations of many countries worldwide, urges research towards more natural and environmentally friendly tools. Among these, chitosan-assisted delivery of biomolecules is a promising method to achieve this goal.

Chitosan (poly(β-(1,4)-2-amino-2-deoxy-*D*-glucose)) is a linear polymer derived from the deacetylation of chitin, the second most available polysaccharide after cellulose [4]. The annual production of chitin from crustaceans, insects, cephalopods, fungi, and algae is around 10 billion tons [5], making chitin and chitin-derived chitosan renewable and ecofriendly materials for a wide range of applications. Chitosan is biodegradable, non-toxic for humans, being considered Generally Recognized As Safe (GRAS) and approved for specific applications [6]. Chitosan-based coatings are widely studied as an alternative packaging for fruit and vegetable to extend their shelf life [7]. Chitosan enrichment with biomolecules such as antioxidants, anti-browning and antimicrobial agents, essential oils, vitamins, plant extracts, and inorganic molecules [7,8,9] can further prolong the conservation, stability, and safety of fresh products. Moreover, the addition of specific molecules in the coating can improve the nutraceutical quality of the food by increasing the concentration of compounds that otherwise would have been present just in trace amounts or absent.

Chitosan is a positively charged molecule and therefore able to interact with the negative charges on the cell membrane [10]. This property makes the chitosan a perfect drug-delivery agent since it can modify the tight junction proteins by interacting with the plasma membrane, thereby inducing the transitory opening of the junctions. This leads to an increase of permeation depending on the degree of deacetylation and molecular weight of the chitosan [10,11]. Moreover, direct interaction of chitosan with phospholipids was reported to increase the membrane permeability of cultured plant cells [12] and induce pore formation in phospholipid vesicles [13]. Thus, it is possible that similar mechanisms (even though plant cells do not possess tight junctions) may allow chitosan-mediated penetration of molecules into fruit flesh.

Tyrosol is a natural phenolic compound whose main sources in the human diet are olive fruit and olive oil. Both the simple phenols tyrosol and hydroxytyrosol, and the main secoiridoid derivatives oleuropein, oleocanthal and oleacein, are well-known health-promoting compounds due to their antioxidant, cardioprotective, antitumoral, anti-inflammatory, and neuroprotective properties [14].

A tomato-based functional food enriched with hydroxytyrosol was produced by adding enzymatically synthesized hydroxytyrosol to tomato juice [15]. However, to the best of our knowledge, no previous work has investigated the enrichment of tomato fruit with tyrosol by the mean of chitosan coating. Moreover, though many papers have been published on coatings enriched with bioactive molecules [16], almost all the works compared the content of the bioactive molecule in coated and control foods without removing the coating before analysis and without studying the kinetics of food enrichment during storage, thus making it impossible to establish if the biomolecule penetrated the peel or was restrained by the coating. In the specific case of fruit, since they are usually washed with water and often peeled before consumption, it is of primary importance to understand not only whether the functional ingredient can migrate from the chitosan film to the fruit but also whether it can cross the peel barrier and accumulate in the flesh.

In the present research, tyrosol was chosen as a model phenolic molecule to study the chitosan-assisted transfer of bioactive compounds into tomato flesh because, although four compounds belonging to the tyrosol family (oleoside dimethylester, oleoside 11-methylester, 3,4-DHPEA-AC and demethyloleuropein) were recently detected in some tomato cultivars [17], its presence in tomato fruit has never been reported.

Based on these assumptions, the present research was addressed to verify

(i) the effectiveness of chitosan coating to transfer bioactive molecules across tomato peel through in vitro tests;
(ii) the possibility of producing tyrosol-enriched tomatoes by post-harvest application of a tyrosol-chitosan coating; and
(iii) the ability of tyrosol to cross the tomato peel also in vivo by measuring the tyrosol content in the fruit peel and flesh separately.

## 2. Materials and Methods

### 2.1. Materials

Tomato fruit (*Solanum lycopersicum* L. var. cerasiforme) were purchased from a local market. They wereselected based on size, color uniformity, and absence of evident signs of damage and carefully washed with water.

### 2.2. Chemicals and Reagents

2-(4-hydroxyphenyl)-ethanol (Tyrosol, TCI Europe N.V., Zwijndrecht, Belgium), 2,2-azino-bis-3-ethylbenzthiazoline-6-sulphonic acid (ABTS), ascorbic acid (Vit. C), potassium persulfate, lycopene, β-carotene, catechin, gallic acid, Folin–Ciocalteu reagent, chitosan, lactic acid, Tween 80 (Sigma Aldrich srl, Milan, Italy). All other chemicals and solvents were of analytical grade.

### 2.3. Preparation of the Formulation

A polymeric dispersion was prepared by adding 1.5% *w*/*w* chitosan to a pre-heated (40 °C) 1% *w*/*w* lactic acid solution, maintained under stirring for 12 h and subsequently introducing 0.1% *w*/*w* Tween 80 [18]. Then, 1% *w*/*w* or 2% *w*/*w* tyrosol was added to the previously prepared dispersion under stirring for 4 h and protected from light to obtain the formulations TYR1 or TYR2, respectively. The amount of tyrosol in the formulation was chosen based on preliminary formulative studies in the range 0.2–5% *w*/*w*. The selection was performed based on physical stability of the formulation and on preliminary permeation results (data not shown).

### 2.4. In Vitro Permeation Study

Tomato peel was separated from the flesh with forceps and utilized for the in vitro permeation studies. They were carried out using Gummer-type diffusion cells consisting of a donor and a receiving compartment between which the excised tomato peel was interposed with an available diffusion area of 1.23 cm^2^; the outer side of the peel faced the donor compartment [19]. The receiving phase (5 mL) consisted of deionized water maintained at 37 °C and stirred at 600 rpm, while 1 mL of TYR1 or TYR2, representative of the donor phase, was placed on the peel surface.

At predetermined time intervals (every 30 min for 5 h), 5.0 mL of the receiving phase were withdrawn for analysis and replaced with the same volume of fresh fluid. The permeation experiment was repeated 6 independent times using the peel excised from 6 individual tomato fruit for each tyrosol formulation.

The quantitative determination of permeated tyrosol was performed by spectrophotometric analysis at wavelength of 275 nm using a Shimadzu UV-2101 PC spectrophotometer.

The concentration of tyrosol in each sample was determined from standard curves obtained by plotting the concentration of known water solutions versus the corresponding absorbance. The calibration curve was linear (r^2^ = 0.999) in the range of 0.687 to 13.74 µg mL^−1^.

Linear regression analysis of pseudo steady-state diffusion plots allowed calculation of the steady-state flux (J), given by Q/A∙t, where Q is the amount of permeant diffusing across the area A in time t, and the percentage of tyrosol permeated at the end of experiment (Q%_5h_) [20].

### 2.5. Improvement of Bioactivity of Tomato Fruit

A total of 160 fruit were washed under current water and randomly assigned to control or chitosan-treated groups. Eighty of them were immersed in the selected formulation (tyrosol-enriched chitosan solution, TYR2) for 15 min and subsequently let dry at room temperature. The remaining 80 untreated fruit were used as control.

Immediately after film formation (T0), a set of tomatoes was thoroughly washed under running water to remove the chitosan coating and, after drying, frozen at −20 °C to preserve the properties until analysis. Other sets of tomatoes were kept at a controlled room temperature with an air conditioner (23–24 °C) for 3, 5, 7 days (T3, T5, T7) before being washed and frozen. Untreated samples at each time were kept as controls. At any sampling time, 10 whole fruit per treatment were frozen whole, while 10 other fruit were peeled with a scalpel, and the peel and flesh of each individual tomato were frozen separately.

The evaluation of fruit bioactivity improvement was performed through different steps: (1) freeze-drying of treated tomatoes, (2) quantitative determination of the amount of tyrosol that penetrated into the tomatoes after appropriate extractive treatment, (3) quantification of the main tomato carotenoids, (4) quantification of total phenolic compounds and flavonoids, and (5) evaluation of the antioxidant capacity of the whole treated tomatoes.

#### 2.5.1. Freeze Drying Process

Exactly weighted tomatoes were cut from frozen into thin slices by the use of scalpels and forceps to avoid touching and then placed in a Petri dish for freeze drying.

The freeze-drying process was carried out in a Virtis apparatus (VirTis Wizard 2.0, SP Scientific, New York, NY, USA) as follows: a freezing phase at −35 °C in 90 min, followed by an extra-freezing phase at −33 °C for 10 min with pressure of 400 Torr; a primary drying phase divided in 5 steps (−35 °C for 20 min at 200 mTorr, −25 °C for 240 min at 150 mTorr, −10 °C for 240 min at 100 mTorr, +10 °C for 300 min at 100 mTorr and +25 °C for 120 min at 100 mTorr); a secondary drying phase at 27 °C for 120 min at 100 mTorr.

Each freeze-dried tomato was extracted by adding 10 mL of deionized water to an exactly weighted amount of tomato (0.3 g), reduced in small pieces; then, the mix was stirred for 24 h in dark conditions. Finally, the dispersions were centrifuged at 4000 rpm for 15 min and the supernatant was collected for analysis. One fraction was used for the quantitative determination of tyrosol by HPLC; the other was subjected to an antioxidant activity assay.

#### 2.5.2. Extraction and Quantification of Tyrosol Amount Penetrated inside Tomatoes

The HPLC analyses were carried out on a Varian Pro Star 330 PDA detector (Varian Corporation, Walnut Creek, CA, USA), equipped with a binary HPLC pump Varian 9012 and a Rehodyne injector with 20 uL loop. Chromatographic separation was carried out by a Luna C18 ODS (Phenomenex 5 μm, 250 × 4.6 mm ID Torrance, CA, USA) HPLC column, protected by a security guard cartridge (Phenomenex C18, 4 × 3.0 mm ID Torrance, CA, USA).

The extracted samples were analyzed according to a procedure previously reported by Gerardi et al. [21] with minor modifications. Chromatographic separation was carried out under isocratic conditions by delivering a solvent mixture consisting of acetonitrile (80%, by volume) and water (20%) by the binary pump at a flow rate of 0.60 mL min^−1^. The HPLC column was kept at a constant temperature of 25 °C and injection volume for all samples and calibrators was set to 20 μL. The DAD detection was performed in the wavelength range from 200 nm to 360 nm and the recording was set at 270 nm for the quantification of tyrosol, which was identified by comparing retention time value and the UV–visible spectra with those of a commercial standard. Quantification was carried out by external calibration.

Approximately 150 mg of freeze-dried material were added to 8 mL of acetonitrile and the mixture was sonicated (15 min, 2 times), vortexed (15 min, 2 times), and centrifuged (15 min, 1500 rpm, 2 times). 6 mL of supernatant were vacuum evaporated, reconstituted with 1 mL of MeOH and filtered through Minisart membranes (0.45 μm pore, Sartorius Stedim Biotech, Goettingen, Germany) before chromatographic analysis.

A solution of tyrosol (1.28 mg mL^−1^) was stored at −20 °C and used as a standard stock solution. The calibration curve was built using 9 standard solutions at the following concentrations: 0.005 (L_1_), 0.01 (L_2_), 0.02 (L_3_), 0.04 (L_4_), 0.08 (L_5_), 0.16 (L_6_), 0.32 (L_7_), 0.64 (L_8_) and 1.28 µg mL^−1^ (L_9_). The linearity interval was determined by adding 100 μL of each tyrosol solution (L1–L9) to 150 mg of freeze-dried material and extracted following the same procedure reported above for the sample preparation.

The areas of the peaks were traced with respect to the known concentrations of standards, and the equation generated, using linear regression, was used to establish the tyrosol concentration in the samples. All the extracts were injected three times and the calibration curve was constructed by plotting the peak areas (y, min mAU) with respect to the concentrations (x, μg mg^−1^). The following straight line (y = 370.74x − 12.29) was constructed from the areas obtained as the function of the tyrosol concentration in relation to the weight of the samples, giving R^2^ = 0.996 in the range of 0.008 to 1.55 μg mg^−1^. The LOD and LOQ were 0.011 and 0.032 μg mg^−1^ respectively and with a recovery of 85%.

#### 2.5.3. Carotenoid Extraction and Quantification

Carotenoid extraction was performed according to Heredia et al. [22], using methanol:acetone:hexane (1:1:1, vol/vol/vol) mix (30 min stirring at 4 °C under dark conditions) and recovering the carotenoid-containing hexane phase after counter extraction with water. Extraction was repeated and the pooled extracts were concentrated by vacuum evaporation. Samples were filtered (0.2 µm PTFE filters, Sartorius Stedim Biotech, Goettingen, Germany) and injected into a HPLC system (P4000 HPLC, UV 6000 LP photodiode array detector, Thermo Fisher Scientific, Waltham, MA, USA). The pigments were separated using a Phenomenex Prodigy LC-18 RP column (5 µm particle size, 250 × 4.6 mm, Torrance, CA, USA) at a flow rate of 1 mL min^−1^ and detected at 445 nm. Lycopene and β-carotene were quantified using calibration curves of commercial standards. Elution was performed using 100% acetonitrile as solution A and methanol:hexane:dichloromethane (1:1:1) as solution B. The elution gradient was 0 min 18% B, 20 min 24% B, 30 min 42% B, 40 min 61% B, 42 min 18% B.

#### 2.5.4. Phenolics and Flavonoid Quantification

Freeze-dried peel and flesh samples were extracted with 80% methanol by a sonication step (30 min), followed by 30 min extraction on a thermo-shaker (BioSan Thermo-Shaker TS-100C, Biosan, Riga, Latvia) at 4 °C. The supernatant was collected by centrifugation (15 min, 14,000× *g*) and the pellet was extracted two other times without the sonication step. The extracts were combined and filtered with 0.2 µm Minisart filters (Sartorius Stedim Biotech, Goettingen, Germany).

Total phenols were determined using the Folin–Ciocalteu colorimetric method as reported by Castagna et al. [23]. Briefly, 1.85 mL of distilled water, 0.125 mL of Folin–Ciocalteu reagent and 0.5 mL of 20% sodium carbonate solution and 25 µL of extract were incubated at room temperature for 30 min. The absorbance was determined versus a blank at 750 nm, and the total phenol content was expressed as mg of gallic acid equivalents (GAE) g^−1^ dry weight.

Total flavonoids were quantified following the method reported by Castagna et al. (2014) [23]. The reaction mix contained 60 µL of 5% NaNO_2_, 40 µL of 10% AlCl_3_, 400 µL of 1M NaOH, 200 µL of water and 100 µL of extract. The absorbance was recorded at 510 nm and flavonoid concentration was expressed as milligram of catechin equivalents (CE) g^−1^ dry weight.

#### 2.5.5. ABTS Radical Scavenging Assay

Stock solutions of 7 mM ABTS and 2.4 mM potassium persulfate were prepared in deionized water. Then, the working solution was obtained by mixing the two stock solutions in 1:1 volume ratio in darkness for 16 h, to reach the end of reaction and to obtain a stable absorbance. Finally, it was diluted (1:10) in water and used for assay. Fresh ABTS solution was prepared for each assay.

100 μL of the tomato extract was let to react with 900 μL of ABTS working solution and, after 15 min, the absorbance at 734 nm was measured. The absence of absorbance at the same wavelength of the tomato extract in the amount used was previously verified.

The ABTS radical scavenging activity, expressed as percentage of inhibition of ABTS radical (I%) was calculated as follow:

I% = [(Abs_ABTS_ − Abs_sample_)/Abs_ABTS_] × 100 (1)

where Abs_ABTS_ is the absorbance of the ABTS radical in water and Abs_sample_ is the absorbance of the ABTS radical solution mixed with a sample extract. All the determinations were performed in triplicate (*n* = 3) [24,25,26,27].

### 2.6. Statistical Analysis

Statistical analysis was performed with GraphPad Prism, version 6.0 (GraphPad Software Inc., San Diego, CA, USA). Statistical differences between permeation parameters were assessed using the Student’s two-tailed unpaired *t*-test (*p* < 0.05). Data are the average of six determinations ± standard error (SE). Differences in the weights of the fruit before and after freeze-drying were assessed using the Student’s two-tailed unpaired *t*-test (*p* < 0.05) on a pool of five determinations for each data set. The influence of storage time on the tyrosol content of the whole tomato, and of fruit peel and flesh separately, was evaluated by one-way ANOVA, followed by the Tukey–Kramer post hoc test (*n* = 3, *p* < 0.05). Significant differences in carotenoid, phenol and flavonoid concentration, and antioxidant activity were checked by two-way ANOVA to evaluate the effect of edible coating and storage time. Separation of means was performed by the Tukey-Kramer test (*n* = 3, *p* < 0.05).

## 3. Results and Discussion

### 3.1. In Vitro Permeation Study

**Figure 1:**
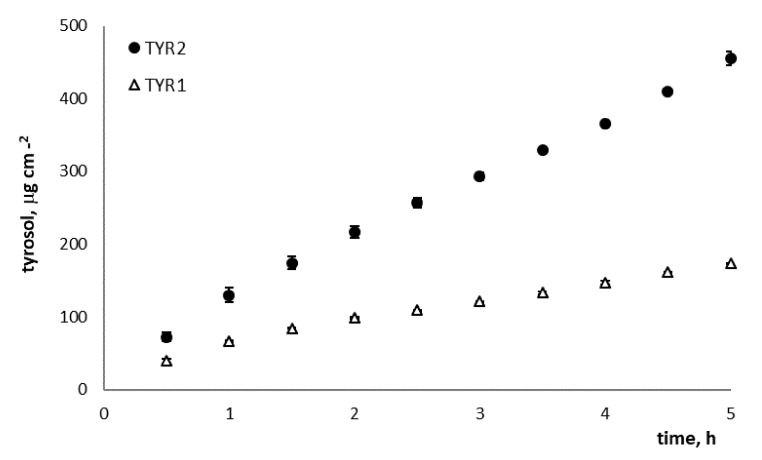
Permeation profile of tyrosol through the tomato peel from the formulations under study, TYR1 and TYR2, 1.0 and 2.0% *w*/*w* tyrosol, respectively (mean ± SE; *n* = 6).

The epicuticolar and intracuticolar waxes of tomato peel represent a barrier for permeation of exogenous hydrophilic substances within the fruit flesh while the presence of polysaccharides and molecules with hydroxyl and unesterified carboxylic groups could promote their permeation [28]. To verify whether the tyrosol molecule was able to permeate across the tomato peel, two chitosan-based formulations (TYR1 and TYR2) containing 1.0 and 2.0% w/w tyrosol, respectively, were tested. The permeation profiles are reported in Figure 1 and the relevant permeation parameters are summarized in Table 1.

The formulation TYR2 produced a 3-fold higher flux through the membrane with respect to the formulation TYR1 containing half the amount of tyrosol (J = 81.57 ± 3.51 and J = 27.88 ± 0.12 µg cm^−2^ h^−1^, respectively) with statistically significant differences. Moreover, the percentage of tyrosol permeated at the end of the experiment was 1.3-fold higher when the formulation TYR2 was applied to the outer side of the peel (3.51 ± 0.30 and 2.73 ± 0.38 for TYR2 and TYR1, respectively). For these reasons, TYR2 formulation was selected for the post-harvesting tomato treatment.

### 3.2. Freeze Drying Process

The amount of tyrosol penetrated into the TYR2-treated tomatoes following the contact with the coating for different periods (T0-T7) was quantified on freeze-dried tomatoes. Moreover, as the coating should delay senescence symptoms on tomato fruit during the post-harvest storage, the water loss expressed as weight loss during fruit storage was measured. No difference in weights between control and treated tomatoes was observed at each time regardless of mincing (Table 2).

In addition, the water percentage inside the tomatoes after the freeze-drying process was calculated. As shown in Table 2, no significant differences in water content (*p* > 0.05) between treated and control tomatoes were observed. Therefore, the chitosan-based edible coating containing tyrosol seemed to preserve the tomatoes from degradation.

### 3.3. Tyrosol Content in Tomato Fruit

**Figure 2:**
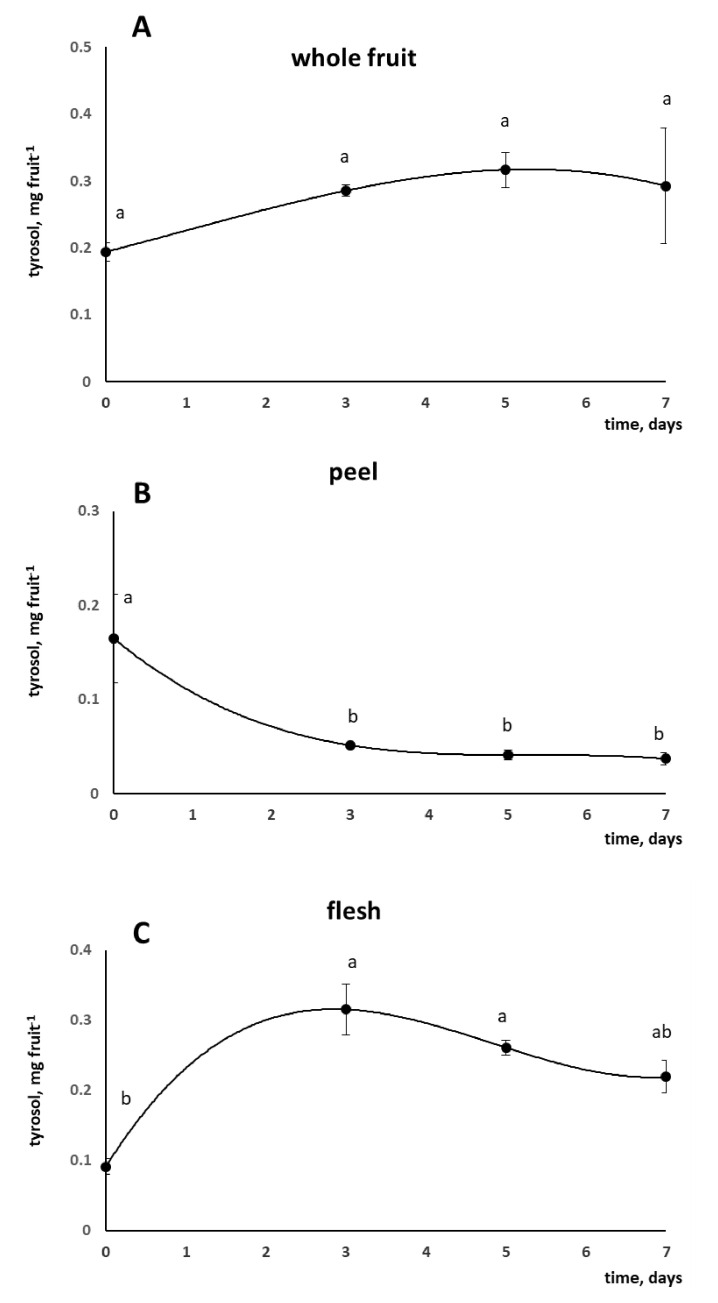
Tyrosol content (mg fruit^−1^, mean ± SE) of whole tomato fruit (**A**), peel (**B**) and flesh (**C**) at different storage times. Different letters indicate statistically significant differences, according to one-way ANOVA, followed by Tukey–Kramer post hoc test (*p* < 0.05).

Tyrosol content was periodically measured in the whole tomato fruit after the application of the coating (Figure 2A). Even though the tomatoes were washed before freezing to allow removal of the tyrosol-enriched chitosan, the presence of tyrosol in the fruit indicated a transfer of tyrosol molecule from the chitosan matrix toward the tomato.

The amount of tyrosol found in the tomato did not differ during storage (*p* = 0.197), suggesting that its release from the chitosan coating reached a threshold value. However, because tyrosol is a powerful antioxidant molecule, the establishment of an equilibrium between its release from the chitosan matrix and its oxidation and degradation during storage cannot be excluded and might explain the invariance of tyrosol content over time.

Based on LARN [29], the daily tomato portion should be 200 g. As we found that about 0.25 mg of tyrosol accumulated in each cherry-tomato fruit (average weight 23 g), about 2.2 mg of tyrosol could be ingested eating one tomato portion. A daily intake of 5 mg of tyrosol, hydroxytyrosol and oleuropein has been approved by EFSA (European Food Safety Authority) because of a health claim about olive oil polyphenols [30]. Such an amount of polyphenols could reduce cardiovascular risk by reducing lipid peroxidation. Therefore, a functional food able to give more than one-third of the recommended daily intake of tyrosol in one portion could be of great interest [30,31,32,33]. It is important to remember that, once ingested, tyrosol can be partially converted to hydroxytyrosol, known for being associated with even more health benefits than tyrosol [14], but, since tyrosol is less prone to auto-oxidation compared to the hydroxytyrosol, it might be more suitable as a stable additive for edible coatings. However, in the light of the slightly bitter and spicy taste of tyrosol, a sensory analysis of tyrosol-enriched tomatoes would be advisable to exclude alteration in the fruit flavour.

In research done by Han et al. [34], the vitamin E content of strawberries (*Fragaria* × *ananassa*) and red raspberries (*Rubus ideaus*) increased following the application of chitosan-based coatings enriched with 0.2% dl-a-tocopheryl acetate. Similar to our finding, these authors observed that vitamin E content did not statistically change in coated berries at the end of two-weeks’ storage although a decreasing trend was evident. However, fruit were analysed without removing the chitosan coating; therefore, it is impossible to establish whether the vitamin E was effectively adsorbed onto the fruit surface or penetrated inside the berries.

Though the application of active coatings enriched with antimicrobial and antioxidant molecules to prolong food conservation and improve quality has been extensively investigated [16], most studies compared the content of the bioactive compound in coated and control food without checking the possible release of the molecule from the coating and its penetration into the food. However, it represents a main concern when an active coating with a specific compound is applied to enrich fruit or vegetables since it could be lost in food washing.

Once it was established that tyrosol could be transferred from chitosan to tomato fruit, the next step was to investigate if it could also pass the peel barrier under in vivo conditions or just remain on the fruit surface. Therefore, the tyrosol content was determined separately in tomato peel and flesh. In the peel, an early significant decrease of tyrosol was observed. Indeed, its concentration was reduced to about one-third after 3 days of storage compared to the initial value (−69%, from 0.165 to 0.052 mg, 0.113 mg lower than T0, Figure 2B). This loss could partially be ascribed to tyrosol oxidation. Simultaneously, tyrosol started to accumulate in the flesh more than it decreased in the peel (+244% at T3, from 0.092 to 0.315 mg, 0.223 mg higher than T0, Figure 2C). This result clearly indicated a centripetal movement of tyrosol from peel to flesh. Moreoever, the tyrosol concentration measured in the flesh after 7 days of storage was still about twice the T0 value, indicating that tyrosol-enriched tomatoes maintain high tyrosol levels during the whole domestic-like storage.

### 3.4. Concentration of Carotenoids, Phenolics and Flavonoids in Peel and Flesh of Tyrosol-Enriched Tomatoes

To check whether the tyrosol-chitosan application affected tomato quality, carotenoids, phenolics and flavonoids concentrations were determined in both peel and flesh during fruit storage.

**Figure 3:**
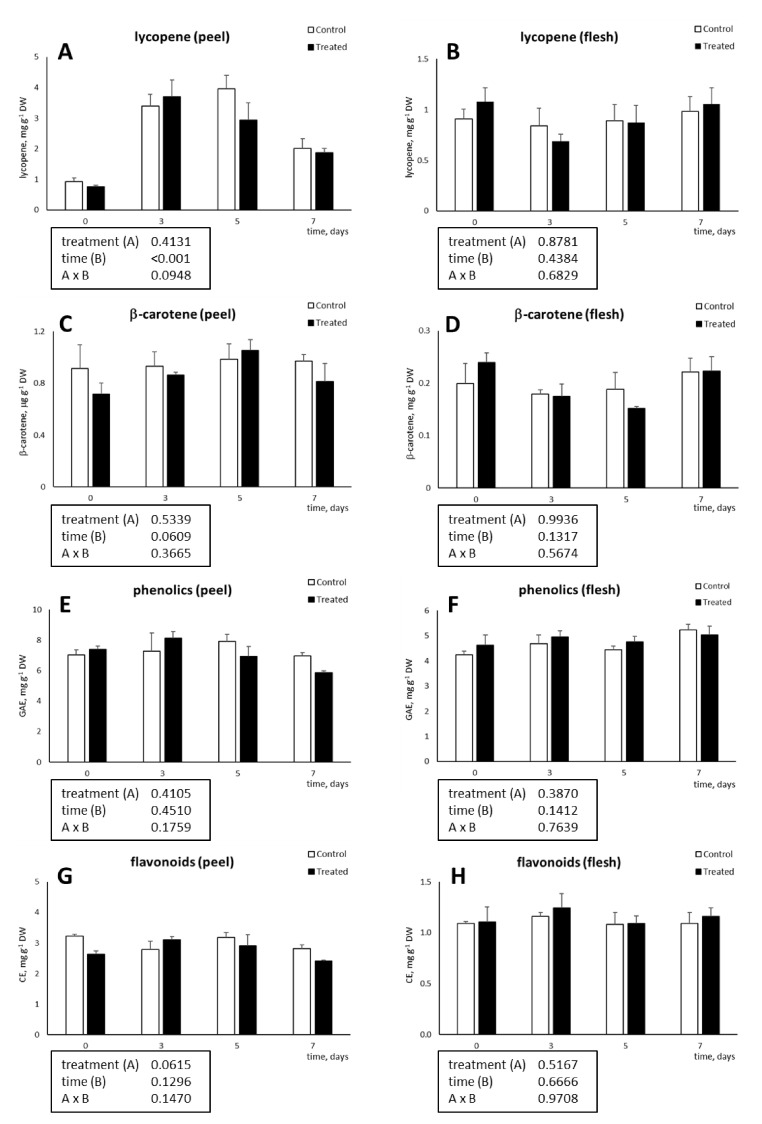
Concentration (mg g^−1^ DW) of lycopene (**A**,**B**) and β-carotene (**C**,**D**) and concentration (mg g^−1^ DW) of total phenolics (**E**,**F**) and flavonoids (**G**,**H**) in peel (**A**–**G**) and flesh (**B**–**H**) of tomato fruit coated with tyrosol-enriched chitosan solution or left uncoated (control) for up to 7 days. The results of two-way ANOVA (*p* < 0.05) are reported in the boxes.

Tyrosol application did not induce any change in lycopene and β-carotene concentrations within the peel (Figure 3A,C). However, a time-dependent enrichment in lycopene occurred from T0 to T3 and T5 due to post-harvest ripening followed by a decrease at the end of the storage period (T7).

In the tomato flesh, both carotenoids were unaffected by the tyrosol treatment, storage time, and interaction between the two factors (Figure 3B,D).

The unchanged carotenoid concentration in both tissues following the tyrosol application suggests that the treatment does not alter the post-harvest quality of the fruit. In previous research, chitosan coating resulted in a lower lycopene concentration after 7 and 11 days of storage, due to a delayed ripening of coated fruit [18]. Such behaviour was not observed in this study, probably because of a more advanced ripening stage of the tomatoes used. Indeed, Pagno et al. [18] observed an increase of lycopene content from day 0 to day 11 due to the progress of ripening, whereas in this experiment, peel lycopene reached its maximum after 3–5 days of storage.

As observed for carotenoids, tyrosol-chitosan coating did not affect the peel and flesh concentration of total phenolic and flavonoids. In both tissues, their levels remained unchanged also during storage (Figure 3E–H).

Phenolic compounds are recognised as highly bioactive molecules, acting as powerful antioxidant and antimicrobial agents, and providing defence against many abiotic and biotic stressors [35]. The finding that the application of tyrosol on tomato fruit did not negatively impact on phenolic and flavonoid content is important to allow the maintenance of a good health status of the fruit during conservation. Due to the above reported properties, phenolics and flavonoids, when ingested with the diet, may play positive roles for human health as well [36,37]. Pagno et al. [18] found that chitosan-coating delayed the loss of some phenolic compounds during tomato storage. Concurrently, the same authors found that other phenolics, such as kaempferol, were more concentrated in control fruit, indicating that chitosan may specifically influence the content of the different phenolic compounds. Accordingly, although in our research no significant differences in total phenolics and flavonoids have been observed, it might be that individual compounds were affected by the treatment.

### 3.5. Antioxidant Activity Assay

**Figure 4:**
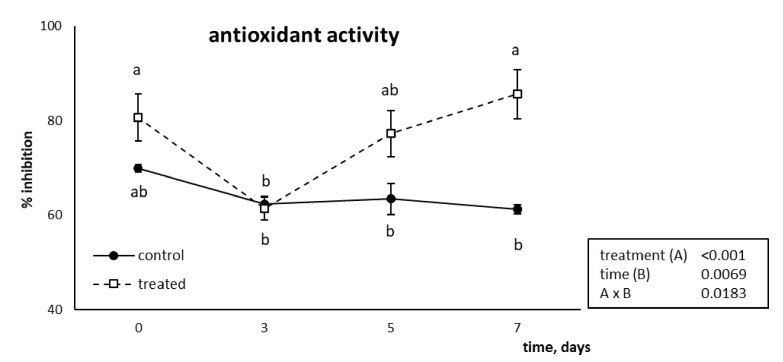
Inhibiting profile of ABTS (mean ± SE) from tomato fruit coated with tyrosol-enriched chitosan solution or left uncoated (control) for up to 7 days. Different letters indicate statistically significant differences, according to two-way ANOVA, followed by Tukey–Kramer post hoc test (*n* = 3, *p* < 0.05). The results of two-way ANOVA are reported in the box.

The antioxidant activity of the tomatoes treated with the TYR2 formulation has been determined to evaluate their behaviour as radical scavengers in comparison with the non-treated controls. The ABTS inhibition profile is reported in Figure 4. While control and treated tomatoes did not show any difference in the antioxidant activity from T0 to T5, with comparable values throughout the storage timepoints considered, at T7 the percentage of inhibition of treated tomatoes increased over control (61.20 ± 0.98 vs. 85.66 ± 5.24 for control and treated tomatoes, respectively).

It is interesting to highlight that, although the amount of tyrosol analytically determined in the treated tomatoes was 11.14 µg mL^−1^, 1.6-fold lower than the minimal inhibiting concentration (17.89 µg mL^−1^) determined through a calibration curve obtained from known concentrations of active compound, the treated tomatoes showed a higher antioxidant activity with respect to the controls. A hypothesis could be that the added tyrosol could progressively replace other phenolics that undergo degradation with time, leading to unchanged total phenolic content but higher antioxidant activity. Moreover, as reported by Pagno et al. [18], chitosan coating itself could have altered the phenolic profile, leading to an increase in the concentration of the most antioxidant compounds and a decrease of the least bioactive ones (e.g., quercetin vs. kaempferol). Finally, a synergistic role played by tyrosol with other antioxidant compounds inside the fruit cannot be excluded.

## 4. Conclusions

The present study demonstrated that tyrosol was able to permeate across the excised tomato peel when applied in a chitosan-based formulation. This finding was further validated by the in vivo application of a chitosan-tyrosol coating on fresh tomatoes. Indeed, tyrosol was found to be retained in the flesh, suggesting a centripetal movement of this compound from peel to flesh has been supposed. Importantly, almost constant levels of tyrosol were maintained up to 7 days of storage at room temperature, indicating the effectiveness of this method to obtain a durable functional food under domestic-like conditions.

The behaviour of tyrosol let us to suppose that other hydrophilic molecules, alone or in combination, could be introduced into fresh products by post-harvest application of chitosan coating.

## Figures and Tables

**Table 1:** Parameters related to permeation of tyrosol from chitosan vehicles through tomato peel (mean ± SE, *n* = 6). Different letters indicate statistically significant differences between formulations, according to Student’s two-tailed unpaired *t*-test (*p* < 0.05). TYR1 and TYR2, 1.0% and 2.0% *w*/*w* tyrosol, respectively.

Formulation	J (µg cm^−2^ h^−1^)	Q%_5h_
TYR1	27.88 ± 0.12 b	2.73 ± 0.38
TYR2	81.57 ± 3.51a	3.51 ± 0.30

**Table 2:** Weights of entire, minced and freeze-dried tomatoes coated or not with TYR2 formulation, and water percentage inside the control and treated tomatoes at different times from treatment with the edible film coating (mean ± SE, *n* = 5).

Sample		Entire Tomato (g)	Minced Tomato (g)	Freeze-Dried (g)	Water (%)
**T0**	**Control**	25.48 ± 3.00	25.41 ± 2.99	1.91 ± 0.30	92.62 ± 0.41
	**Treated**	20.93 ± 1.41	20.90 ± 1.41	1.74 ± 0.08	91.60 ± 0.25
**T3**	**Control**	26.27 ± 1.90	26.24 ± 1.89	2.07 ± 0.18	92.12 ± 0.34
	**Treated**	22.63 ± 1.46	22.57 ± 1.45	1.82 ± 0.08	91.90 ± 0.27
**T5**	**Control**	24.98 ± 3.03	24.92 ± 3.03	1.93 ± 0.19	92.07 ± 0.62
	**Treated**	23.98 ± 2.32	23.90 ± 2.31	1.99 ± 0.21	91.66 ± 0.28
**T7**	**Control**	24.52 ± 2.08	24.48 ± 2.07	1.97 ± 0.15	91.92 ± 0.28
	**Treated**	24.27 ± 2.60	24.23 ± 2.61	1.87 ± 0.25	92.30 ± 0.31

## Data Availability

The data presented in this study are available on request from the corresponding author.
